# Identification of significant proxy variable for the physiological status affecting salt stress-induced lipid accumulation in *Chlorella sorokiniana* HS1

**DOI:** 10.1186/s13068-019-1582-9

**Published:** 2019-10-12

**Authors:** Seung Hwan Oh, Yong Keun Chang, Jay Hyung Lee

**Affiliations:** 1 Department of Chemical and Biomolecular Engineering, KAIST, 291 Daehak-ro, Yuseong-gu, Daejeon, 305-701 Republic of Korea; 2 Advanced Biomass R&D Center, KAIST, 291 Daehak-ro, Yuseong-gu, Daejeon, 305-701 Republic of Korea

**Keywords:** Microalgae, *Chlorella sorokiniana* HS1, Two-stage cultivation, Stress-induced lipid accumulation, Physiological status

## Abstract

**Background:**

Current efforts on the optimization of the two-stage cultivation using stress-induced lipid accumulation have mostly focused only on the lipid induction stage. Although recent studies have shown that stress-induced lipid accumulation is affected by the physiological status of the cells harvested at the preceding cultivation stage, this issue has hardly been examined hitherto. Such a study needs to be carried out in a systematic way in order to induce lipid accumulation in a consistent and predictable manner with regard for variances seen at the cultivation stage.

**Results:**

After a photoautotrophic cultivation of *Chlorella sorokiniana* HS1 in a modified BG11, harvested cells were re-suspended in the fresh medium, and then NaCl was added as the sole stress inducer with light illumination to induce additional accumulation of lipid. Effects of culture temperature on the lipid accumulation were analyzed by the Kruskal–Wallis test. From the microscopic observation, we had observed a definite increase in lipid body induced by the stress since the cell entered a stationary phase. A multiple linear regression model was developed so as to identify significant parameters to be included for the estimation of lipid induction. As a result, several key parameters at the end of cultivation, such as cell weight, total lipid content, chlorophyll *a* in a cell, and Fv/Fm, were identified as the important proxy variables for the cell’s physiological status, and the modeling accuracy was achieved by 87.6%. In particular, the variables related to Fv/Fm were shown to have the largest influence, accounting for 65.7% of the total variance, and the Fv/Fm had an optimal point of maximum induction at below its average. Clustering analysis using the *K*-means algorithm indicated that the algae which are 0.15 pg cell^−1^ or less in chlorophyll concentration, regardless of other conditions, had achieved high induction results.

**Conclusion:**

Experimental results showed that it usually achieves high lipid induction after the cells naturally end their division and begin to synthesize lipid. The amount of lipid induction could be estimated by the selected proxy variables, and the estimation method can be adapted according to practical situations such as those with limited measurements.

## Background

A well-known strategy for raising the lipid content of a microalgal cell is the cell stress-induced lipid accumulation, which has the advantage of being relatively simple to implement and therefore readily applicable to most microalgae cultivation processes. Salt addition, which applies cell stress through osmotic pressure, has been demonstrated in practice as a viable means to increase the lipid content in microalgae [1, 2]. Nevertheless, it is known that such cell stress conditions inhibit cell division and are usually unfavorable for biomass production [3–6]. It is well known that energy storage in microalgae is coupled with regulation cell division [5, 6]. In other words, stress-induced lipid accumulation alone will not be suitable for increasing the lipid production in general. As a solution, two-stage cultivation has been proposed and received much attention recently [1, 2, 7–12]. The two-stage cultivation comprises the biomass production stage (BPS) and the lipid induction stage (LIS) performed in sequence, to achieve high lipid content without lowering biomass productivity. In the BPS, microalgae are firstly grown in a growth-supported condition, and then are transferred to a growth-limited condition to induce lipid accumulation within the grown cells.

Previous work on optimization to maximize lipid production in the two-stage cultivation has mostly focused on the induction condition applied at the LIS such as the induction time and stress level [2, 7, 11, 12]. In comparison, the effect of the culture conditions (e.g., temperature, light intensity) of the BPS on the subsequent lipid induction is still poorly understood. Recent research works indicate strong evidence that cell’s physiological status, manifested by the culture condition of the BPS, highly affects the lipid induction result [4, 9, 10]. Some studies on determining the optimal harvesting time for lipid induction were carried out using lipid productivity and lipid content as the criteria [1, 8]. However, these studies also did not take into account the cell’s physiological status after the BPS in the optimization. To the best of our knowledge, there has been little study that considered the physiological status of the harvested cell in the optimization of lipid induction. As the lipid synthesis mechanism of microalgae is very complicated and sensitive to cell’s physiological status, it can be expected that the final lipid content after the LIS would vary depending on the harvested cell’s condition even with identical salt stress. In semi-continuous cultivation, TAG production using nitrogen starvation in *Nannochloropsis oceanica* IMET1 was successfully optimized by regulating intracellular nitrogen, and ΔF/Fm′ was used as a proxy variable for estimating intracellular nitrogen [4]. The study was performed under well-controlled culture conditions that were difficult to control in outdoor cultivation, but showed that quantitative control of lipid production could be achieved by monitoring cell status.

In order to reduce cultivation cost, in practice, microalgae are frequently grown outdoors using inexpensive fertilizers and flue gas. Due to the inconsistent compositions of these raw feed materials, significant variabilities are observed in the final quality of biomass. Furthermore, diurnal variations in outside weather condition (e.g., temperature, light intensity) make it difficult to maintain biomass quality. Nevertheless, it is hard to justify additional spending for ensuring biomass quality, e.g., to implement a heat exchanger or supplementary light, as the high production cost of microalgae is the main obstacle to commercialization [13]. The variation in biomass quality not only makes the lipid induction result inconsistent but also presents a major problem in subsequent biofuel refining processes. Retrofitting of existing petroleum refinery for bio-based raw material can reduce the size of initial capital investment needed. In converting existing refineries to bio-refineries, however, significant engineering challenges arise due to the large variations in bio-based feedstock quality. Petroleum feedstocks also show variations in composition, but they are relatively smaller than in biomass, and many existing refineries are not designed to handle such wide variations in biomass feedstock [14].

In the situation where the quality of biomass is not consistent and the induction results vary significantly, the use of the stress-induced lipid accumulation presents the risk of further increasing the variabilities to the refinery process. However, if the amount of lipid induction can be effectively controlled despite the variations in the incubation condition and raw material consumed, the lipid induction can be worthy of consideration. So, it is required to understand how the physiological status of the harvested cell affects the lipid induction performance in order to make the induction outcome more predictable and eventually consistent. As proved by the previous studies [4, 9, 10], the correlation between the cell’s physiological status and the lipid induction result was evident, but this issue had hardly been examined before. Even the previous studies that observed the very effects have not suggested a quantitative indicator or proxies that can be used to determine an appropriate time for harvesting (for the subsequent application of stress-induced lipid accumulation) other than mentioning that stress should be applied at an appropriate time [9, 10]. Consequently, for the *Chlorella sorokiniana* HS1, accumulating lipid under salt stress, optimal salt concentration varied with the incubation condition even for the identical strain [2, 12]. Thus, we should identify appropriate measurable proxy variables for the cell’s physiological status strongly related to the lipid induction result including growth phase.

In this study, proxy variables for the cell’s physiological status with strong relationships with the lipid induction result are selected. As a practical guideline, the measurement priority and its recommended level for harvesting are given. This study is carried out in the following sequence: First, two-stage cultivation of *Chlorella sorokiniana* HS1 strain is performed using NaCl as a stress inducer. Microalgae samples with various physiological statuses are created by varying the culture condition and harvesting period. Next, significant proxy variables are selected using the method of all possible regressions which examines all the combinations of the following parameters of the harvested cell after the BPS: cell weight, total lipid content, non-starch carbohydrate content, starch content, chlorophyll *a* in a cell (Chl *a*), and maximum quantum yield of PSII (Fv/Fm). Finally, *K*-mean clustering is carried out in order to determine which parameter among the proxy variables should be measured as a high priority to achieve stable lipid induction results when accurate estimation of all the relevant variables is difficult due to limitations in measurement.

## Results and discussion

### Influence of cell condition on lipid induction

**Fig. 1:**
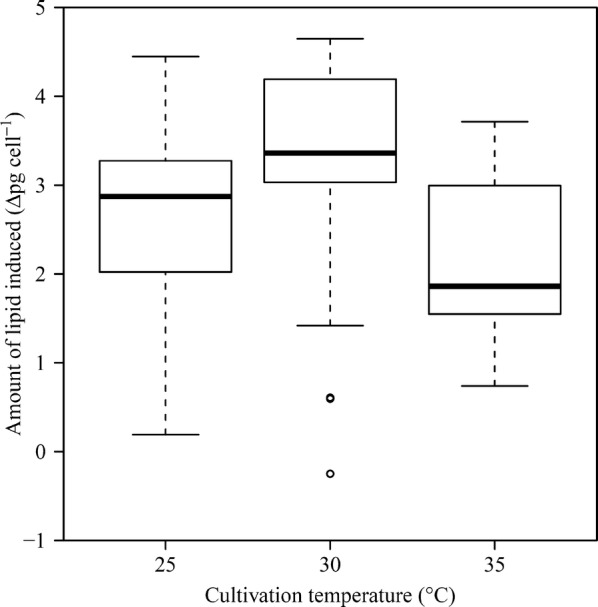
Influence of culture temperature at the BPS on lipid induction at the LIS. Whiskers indicate the minimum and maximum values of the groups. The central box represents the values from the lower to the upper quartile (25 to 75%), and the middle line depicts the median. Values beyond the quartiles by one-and-a-half of the inter-quartile range are depicted as open circles

In order to investigate the difference in the lipid induction results due to the incubated cell’s physiological status, microalgae samples of various conditions were created (Additional file 1: Table S1). Definitely, the physiological status of the cell can be changed by various factors, such as pH and light intensity; meanwhile, the study focused on the effects of temperature and growth phase, one of the most influential factors in outdoor culture. Predetermined optimal salt stress (40 g L^−1^) for 24 h was identically applied to all cultures to distinguish the effects of cell’s incubation condition from the influence of salt stress variation (Additional file 2: Figure S1). In order to increase the statistical significance, samples were incubated independently with random order and remaining cells were discarded entirely except for the cells to be used for lipid induction. In Fig. 1, the amount of lipid induced vs. culture temperature at the BPS was plotted using a box plot. The Shapiro–Wilk test was performed to check the normality in the distribution of each group [15], and the homogeneity of variance was assessed by the Levene’s test. All groups met the assumption of equal variances, but samples cultivated at 30 °C failed to satisfy the normality condition. This is due to the fact that the number of high induction sample was greater than a low induction (data skewed to the left). Thus, the Kruskal–Wallis test was used to compare the differences in the induction level [16]. As a result, the effect of the incubation temperature at the BPS yielded a Chi-squared value of *χ*^2^(2) = 6.584, *p* < 0.05, indicating that the incubation temperature had a significant effect on the lipid induction. Depending on the temperature, the proxy parameters for physiological status of the harvested cells were very different (Additional file 3: Figure S2). Since the incubation temperature corresponds to a cause for the changes in the harvested cell’s physiological status, it is reasonable to say that the status of the cell rather than the culture temperature itself is closely related to the subsequent lipid accumulation behavior.

Among the measured parameters, chlorophyll content was found to have the highest correlation with lipid induction based on the Pearson correlation, which gave: cell weight (− 0.08), total lipid content (0.24), non-starch carbohydrate content (− 0.17), starch content (0.42), Chl *a* (− 0.60), and Fv/Fm (− 0.42). This result agrees with the observations that the 30 °C culture group which had the lowest chlorophyll had achieved the largest induction level of 3.07 Δpg cell^−1^. Prior to the nutrient deficiency, the triacylglyceride (TAG) stored inside the cell is consumed rather than accumulated because the energy gained by photosynthesis is less than the energy required for cell division [17]. When nutrients in the medium become insufficient, anabolic processes such as cell division will be disturbed in need of these nutrients [3, 18]. As the rate of anabolism slows, more energy is produced by photosynthesis than consumed by the anabolic process, and some of the excess energy is converted to TAG and stored in the cell. This synthesized TAG is utilized for cell growth when growth-supported environments are restored [19, 20]. At the same time, to alleviate the stress caused by the excess energy, microalgae reduce the degree of photosynthesis by changing the concentration of chlorophyll according to the external nutrient conditions [21]. As a result, the Chl *a* decreases gradually as the external nitrogen sources are consumed. For this reason, chlorophyll content can be an index to represent how much lipid can be synthesized once the cell is put under stress and the lipid synthesis starts dominating over the cell division. Similarly, the Fv/Fm could be another such index because it also decreases with the generation of excess energy which implies lowering quantum efficiency [22]. It is well known that starch is the primary storage component, and it can be converted to lipid, and *C. sorokiniana* HS1 synthesized lipid subsequent to a degradation of stored starch under the nutrient-deficient condition [23–25].

**Fig. 2:**
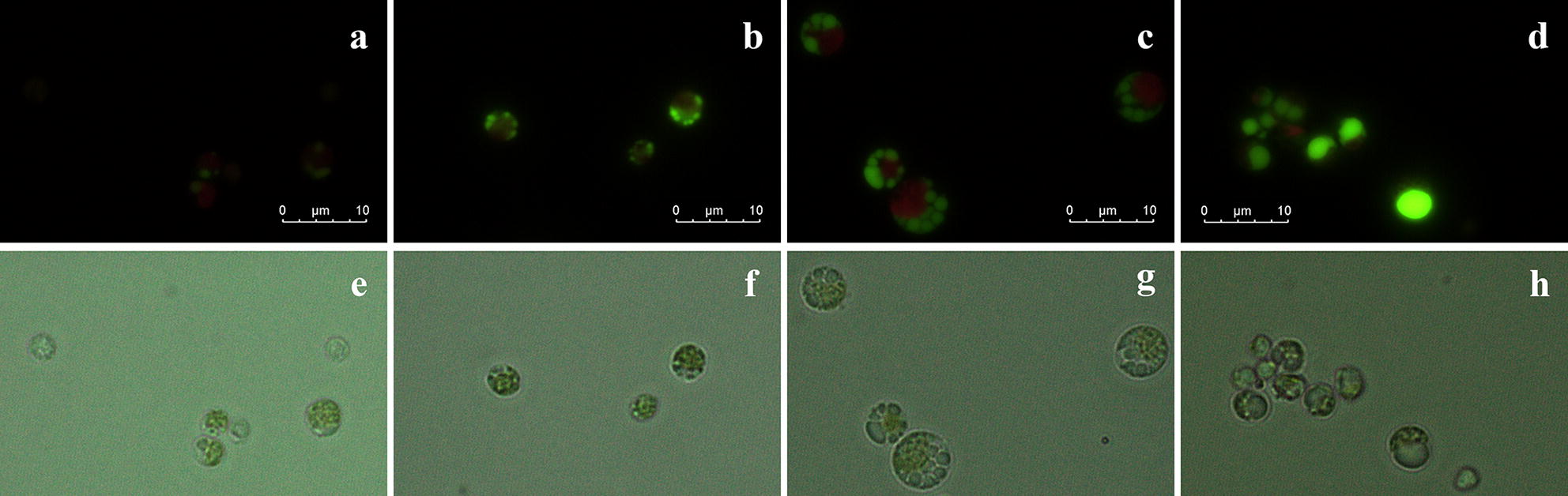
Effect of the harvesting period on the volume and morphological changes in the lipid droplets. Algal cell stained with BODIPY 505/515 and viewed under fluorescence microscopy with a 450–490 nm band excitation filter and a 515-nm-long emission filter (**a**–**d**). Fluorescence of the dye in the presence of cellular lipids can be seen as green fluorescence. In microalgae, autofluorescence of chlorophyll is also observed with red fluorescence. Micrograph of microalgal cells under bright field (**e**–**h**). Microalgal cells were cultured for 2 days (**a**, **e**), 4 days (**b**, **f**), 10 days (**c**, **g**), and 14 days (**d**, **h**). The cells were incubated at 30 °C in the BPS and underwent 48 h of lipid induction

Images of *C. sorokiniana* HS1 were obtained to observe changes in the morphology of lipid droplets after the LIS (Fig. 2). The identical stress of 48 h of induction was applied to the cells harvested at the different times and then stained by the green fluorescent dye, BODIPY 505/515 (4,4-difluoro-1,3,5,7,-tetramethyl-4-bora-3a,4a-diaza-s-indacene). In the figure, cells that were harvested early (on day 2) did not have distinct lipid droplets (Fig. 2a). In the images taken from the bright field, chloroplasts appeared to have lost their photosynthetic function due to the excessive stress (Fig. 2e). Considering the fact that synthesis of lipids under a salinity condition is a protective mechanism to protect the microalgae from the stress [1], they seemed to be less capable of protecting themselves. Likewise, Mus et al. [10] observed similar results that a larger increase in lipid content resulted with an addition of bicarbonate in *Phaeodactylum tricornutum* culture, but this addition had a positive effect only when it was made on a fourth day (aged cell) and not at the beginning. As the exponential phase began in earnest (day 4), distinguishable lipid droplets started to appear inside the cell (Fig. 2b). Since then, huge lipid droplets were being accumulated after the cell reached the stationary phase (Fig. 2c, d). The optimal growth temperature of *Chlorella sorokiniana* HS1 is about 30 °C, and as shown in Fig. 1, samples grown at 30 °C did not satisfy normality due to the bias of the data; these results suggest that the stationary phase is favorable for lipid accumulation. The excess energy can be utilized to synthesize useful lipids for future cell growth or maintaining the cell wall’s fluidity to endure cell size expansion under hypertonic solution. However, it was evident that lipid induction varied depending on the physiological status of the cell, and significant induction was possible only when the microalgal cells were sufficiently mature to synthesize lipid form the excess energy.

### Development of a predictive model to identify significant parameters

Without knowledge about physiological status which can affect the lipid accumulation, it is hard to decide which variable must be controlled to induce for the desired level of lipid induction. The significance of each variable was evaluated based on its contribution to the explanatory power to estimate the amount of lipid induction of a predictive multiple linear regression (MLR) model which is the one of the basic machine learning techniques [26]. This influence of each parameter on the model’s prediction capability was evaluated by the relative importance, which quantifies an individual variable’s contribution to a MLR model [27].

The predictive model was developed by MLR which has proven effective in many biofuel applications, such as an application to estimate bio-crude yield in a microalgae hydrothermal liquefaction process [28–30]. In this study, the six intrinsic variables (cell weight, total lipid content, non-starch carbohydrate content, starch content, Chl *a*, and Fv/Fm) were considered as candidate variables. All the data were standardized before the regression, obtained by subtracting the mean from the variable and dividing by the standard deviation so that each independent variable has zero mean and unit variance.

**Fig. 3:**
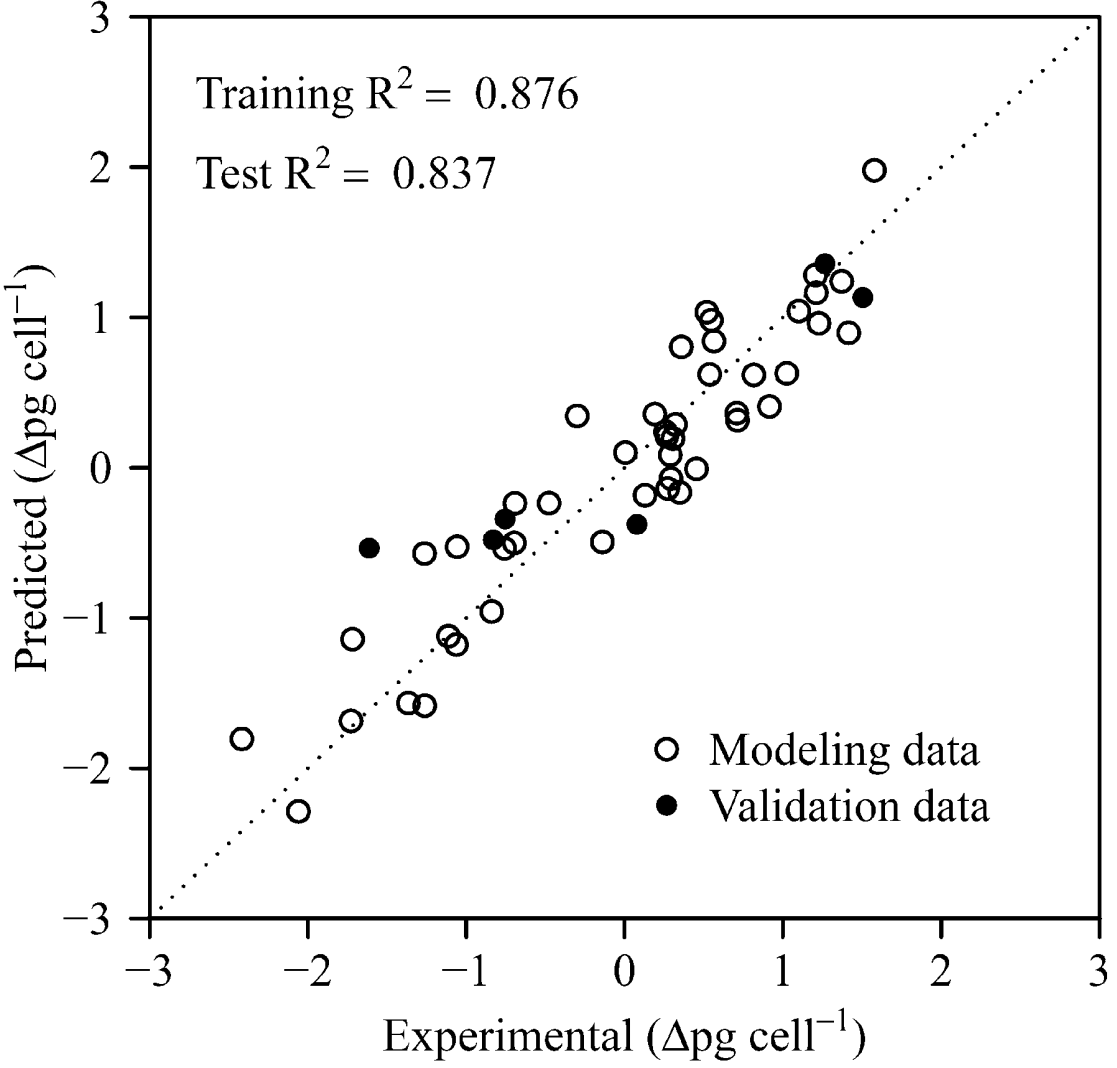
Results of the best regression model for the prediction of the amount of lipid induced. The samples were plotted in the standardized unit

Of the six variables, the model containing the cell weight, total lipid content, Chl *a* and Fv/Fm in the regressor was chosen as the best model. Detailed model information such as the variable coefficients is represented in Table 1. All the parameters were statistically significant including the quadratic (denoted by superscript) and interaction (denoted by a colon) term. The regression coefficients shown in the table are the standardized coefficients, which are the results of regression analysis with standardized data. As shown in Fig. 3, the implemented MLR model predicts the model training data (*R*^2^ = 0.876) as well as the test data (*R*^2^ = 0.837) very well.

**Table 1:** Influence of the regressor variables on the amount of lipid induced

Regressor^a^	Coefficient		*t* value	*p* value
	Estimated	Standard error		
Intercept	0.820	0.093	8.87	< 0.001
CW	− 0.754	0.090	− 8.41	< 0.001
L	0.167	0.079	2.11	0.042
CH	− 0.195	0.081	− 2.41	0.021
FV	− 0.859	0.111	− 7.71	< 0.001
L^2^	− 0.251	0.047	− 5.38	< 0.001
FV^2^	− 0.560	0.070	− 7.96	< 0.001
CW:FV	0.514	0.104	4.94	< 0.001
L:FV	− 0.620	0.137	− 4.53	< 0.001

*CW* cell weight, *L* total lipid content, *CH* Chl *a*, *FV* Fv/Fm

^a^Data were centered and scaled before modeling

**Fig. 4:**
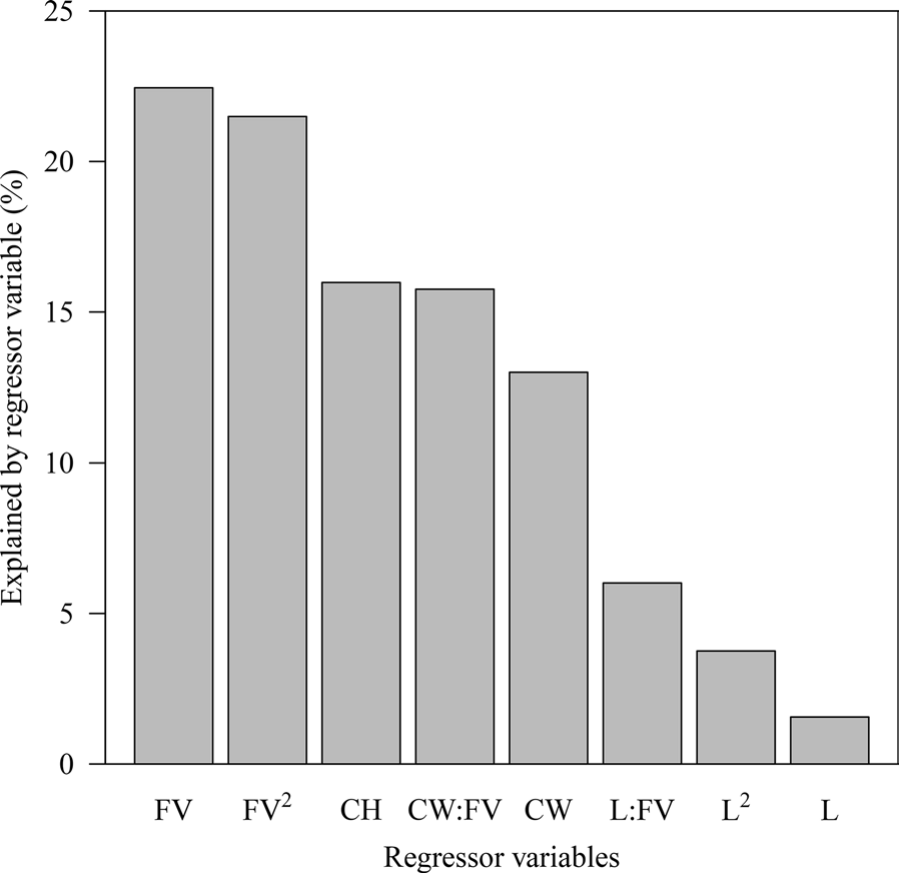
Relative importance of the regressor variables. Metrics are normalized to sum to 100%. *CW* cell weight, *L* total lipid content, *CH* Chl *a*, *FV* Fv/Fm

The relative importance for each parameter was investigated (Fig. 4). The Fv/Fm parameter was the most involved one in explaining the changes in lipid induction (22.4%), followed by its quadratic term (21.5%). In the case of chlorophyll which had the third highest explanatory power (16.0%), only the main effects existed without any interaction or quadratic term. It was followed by the interaction term between cell weight and Fv/Fm (15.8%) and the main effect of cell weight (13.0%), and all the parameters containing lipid content had a descriptive power of not exceeding 10% (6.01, 3.75 and 1.56%, respectively). The Fv/Fm parameter was used as the most important variable in the prediction of lipid induction to the extent that it was included in half of all parameters. Cell weight was less informative than chlorophyll, but there was a strong interaction with Fv/Fm, as the effect of cell weight on lipid induction changed significantly according to the level of Fv/Fm. In conclusion, measurement of Fv/Fm was essential for predicting the lipid induction behavior, and chlorophyll and cell weight were also helpful for accurate predictions. Lipid content would be the first to go if some of the parameters were to be removed from the candidate list.

### Physiological significance of the regressor variables

The information about the selected feature and its relative importance could give us qualitative knowledge about the microalgal cell’s state for optimal lipid accumulation. The physiological status of the lipid induction stage was not analyzed since the purpose of the study is to predict lipid induction based on the status at the time of harvest. The *C. sorokiniana* HS1 has pathways to synthesize TAG de novo through the external carbon assimilation or utilizing the internal pre-formed starch [2]. Whichever pathway is activated, energy is required to convert the carbon sources into lipid. Since the green microalgae obtain the necessary energy through photosynthesis, lipid accumulation behavior is bound to be affected by the level of Fv/Fm. From the above model, the Fv/Fm had negative coefficients for both the linear and quadratic terms (Table 1). If the quadratic term has a negative coefficient, the function is plotted in a downwardly convex form. Furthermore, its vertex is located at a point less than zero if the signs of the quadratic and linear coefficients are the same. Since all the data used were standardized (with zero mean value), these two parameters suggested that the optimal point where the lipid induction amount is the largest is at a value lower than the average Fv/Fm value. This means that a lower Fv/Fm value generally meant a high lipid induction amount but only to a point, and if the harvested cell showed an Fv/Fm value even lower than the optimal point, it would give a worse induction result. The Fv/Fm is one of the most reliable proxy indicators to quantify the cell’s physiological status in relation to its health, and as the cell ages, its value decreases due to the cell becoming less photosynthetically active and less efficient [31]. Given that lipid is predominantly synthesized through the excess energy generated by the reduction in photosynthetic efficiency, the decrease in Fv/Fm means more active lipid synthetic mechanism and aging of the cells at the same time. In conclusion, it was favorable to accumulate lipid when the microalgae were in a balanced state in terms of aging. The largest relative importance of Fv/Fm means that an appropriate balance in cell maturity affects the lipid induction result the most (Fig. 4). However, the high importance of the interaction term involving Fv/Fm implies that the induction mechanism indirectly represented by a simple quadratic function of the Fv/Fm is not enough (Table 1).

The cell weight, which is proportional to cell size, could be another important variable considering the globular morphology of *Chlorella sorokiniana* HS1. As the cell surface area decreases, transport through the cell membrane decreases, thereby lowering the rate of obtaining nutrients for lipid synthesis from the medium. On the other hand, reduced surface area alleviates the osmotic stress the cell receives. The negative coefficient of the main effect of the cell weight means that the smaller the cell size, the better the lipid accumulation, which suggest that the stress the cell receives rather than the absorption rate of absorption of nutrients determines the lipid induction. In addition to the variation due to cultivation condition, the cell size may vary depending on the growth phase. For example, small cell size usually corresponds to an exponential growth phase in which cell division is highly activated, and the cells at this period are not yet suitable for lipid accumulation (Fig. 2). But obviously, the positive effect of small cell size in the early stages of growth, which is undesirable in fact, is limited by the interaction between cell weight and Fv/Fm. The interaction term had a positive sign, which represents that lipid induction was compromised for the infant cells that lack resistance to salt stress, even if the cell size was smaller than the mean (higher Fv/Fm value than the mean).

The improvement in lipid induction for the matured cell also can be seen through another indirect indicator, the chlorophyll concentration, which has a negative coefficient in the regression model. It is well known that lipid accumulation in oleaginous microalgae is coupled with the degradation of chloroplast under stress condition [19]. When the cells stop growing and enter a stationary phase under nitrogen starvation, the useless chloroplast is degraded and stored into the lipid body [18]. It is notable that unlike Fv/Fm, Chl *a* did not have any quadratic or interaction term. This is due to the fact that there is not much difference in the chlorophyll concentration after nitrogen starvation, compared to Fv/Fm which continues to decrease as the cultivation progresses (Additional file 1: Table S1). Hence, the model parameter is relatively less sensitive to the chlorophyll than the Fv/Fm after the cells entered the stationary phase.

Proper salt addition increases the lipid content of microalgae, but excessive stress suppresses lipid accumulation [32]. Under salt stress conditions, lipids have the ability of cells to tolerate osmotic stress by maintaining fluid in the cell membrane and reducing the deformation of cells due to osmotic pressure [1]. Thus, lipids can alleviate excessive stress, reducing the inhibition of photosynthesis required for TAG accumulation. The total lipid content yielded quadratic and main effect term, but they had different signs, which meant that the optimum point is located above the mean. The interaction terms implied that the lipid also had complex correlations with Fv/Fm, but the effect was not significant in that the relative importance of all the terms for the lipid content combined was less than that of the chlorophyll’s main effect. This result implies that the key point is the balance of photosynthesis and TAG synthesis, and factors related to photosynthesis such as Fv/Fm and Chl *a* seem to reflect this phenomenon better.

### A harvesting strategy when measurement is limited

Although it is best to determine the harvesting state of the microalgae based on the regression model, measurements of some of the involved variables may be limited due to the maintenance and cost issues. Hence, a simpler strategy that does not require expensive and time-consuming measurements may be more practical even if the accuracy of the prediction is compromised a bit. If the purpose is to decide when to harvest the microalgae at the BPS rather than an accurate prediction of the amount of lipid induced after the LIS, classification instead of regression can be used to identify the group with high lipid induction amounts. Thus, the index parameters were investigated by testing the clustering performance of all possible subsets of them.

The *K*-means clustering algorithm which is one of the basic machine learning techniques was used as the classification method, and the silhouette score was used for the evaluation criteria. Since the purpose of classification is to identify status that facilitates the lipid accumulation, there should be a notable distinction in the amount of induction between the groups, in addition to their physiological status. Thus, in addition to the selected proxy variables, the amount of lipid induced was included in all the clustering subsets to increase the classification performance with respect to the lipid induction behavior, and the silhouette score was calculated using the amount of lipid induced only. The silhouette score was calculated for all possible parameter subsets by varying the number of clusters (Additional file 4: Table S2). The combinations with the highest silhouette score for each number of the variables used are shown in Table 2. Regardless of the number of the variables used in the classification, the results showed a division into three distinct groups. The Fv/Fm which described the variation the best in the regression model was included in subsets with two or three variables. On the other hand, when only one variable was used for clustering, Chl *a* was chosen as the representative variable instead of Fv/Fm. In the correlation analysis, the chlorophyll was the most correlated variable with the lipid induction. The Fv/Fm was also highly correlated, but it had less linear interpretability than the chlorophyll due to its nonlinear effects. As seen in the MLR, the influence of the Fv/Fm has complex behavior which varies depending on the state of the cell (Table 1). While it is complicated to analyze the effect of Fv/Fm without information about the other variables, the Chl *a* had an obvious negative relationship with the amount of lipid induced with just the main effect.

**Table 2:** Optimal clustering result for each number of the proxy variables used

Number of proxy variable^a^	Selected variable	Number of cluster	Silhouette score
3	CW, CH, FV	3	0.251
2	CH, FV	3	0.274
1	CH	3	0.358

*CW* cell weight, *L* total lipid content, *CH* Chl *a*, *FV* Fv/Fm

^a^Data were centered and scaled before modeling

**Fig. 5:**
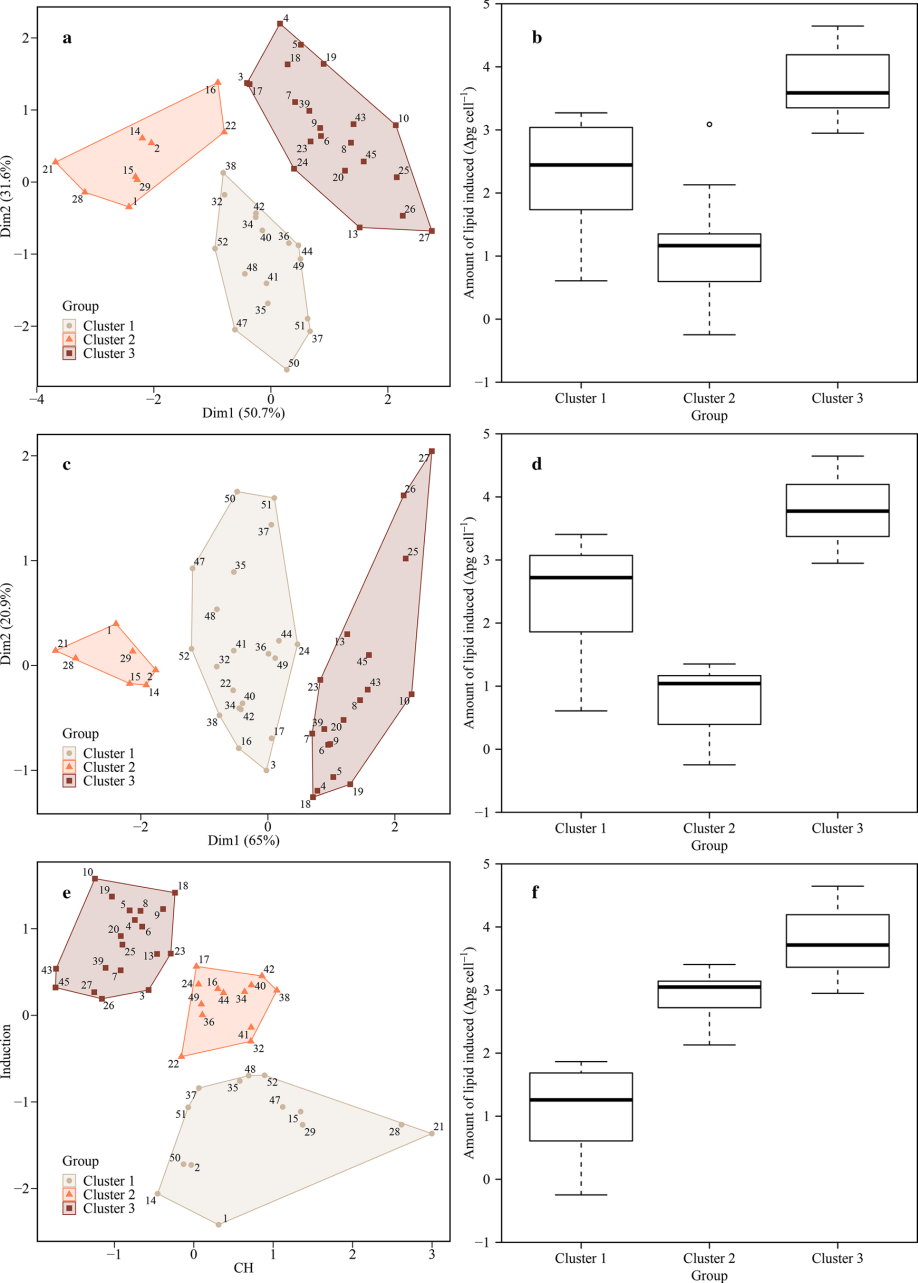
*K*-means clustering result with the best classification performance for each number of the variables used. Biplots consisting of the principal components are used to indicate the distribution of clustered samples (**a**, **c**, **e**), and the corresponding quantities of lipid induction amount for each cluster are expressed in boxplots (**b**, **d**, **e**). In the case of just one variable used, the axes represent the corresponding condition and the lipid induction (**e**). Three conditions (**a**, **b**), two conditions (**c**, **d**), and one condition used (**e**, **f**). CH Chl *a*

In Fig. 5, the clustering results for the best subsets are shown. Partitioning of each group is visualized using the principal components as axis labels (Fig. 5a, c) and plotted with an intrinsic variable and the lipid induction amount as axis labels when only one variable was used (Fig. 5e). Each observation was represented as a point labeled by its experiment number in the plot (Additional file 1: Table S1). In all the clustering results, each group has a distinct lipid induction distribution (Fig. 5b, d, f). It is notable that the distinguishability for the physiological status to induce the highest lipid accumulation was not that different with the Chl *a* alone, compared to the cases when several variables were used. Besides, the average induction in the most induced group was higher than in the case that cell weight, Chl *a*, and Fv/Fm were all used, with almost no difference in the standard deviation (Additional file 5: Table S3). In particular, it has the advantage of being simple and intuitive: Lipid accumulation increases as chlorophyll contents decrease. This can lead to an easily implementable protocol with a simple measurement requirement and interpretation.

**Fig. 6:**
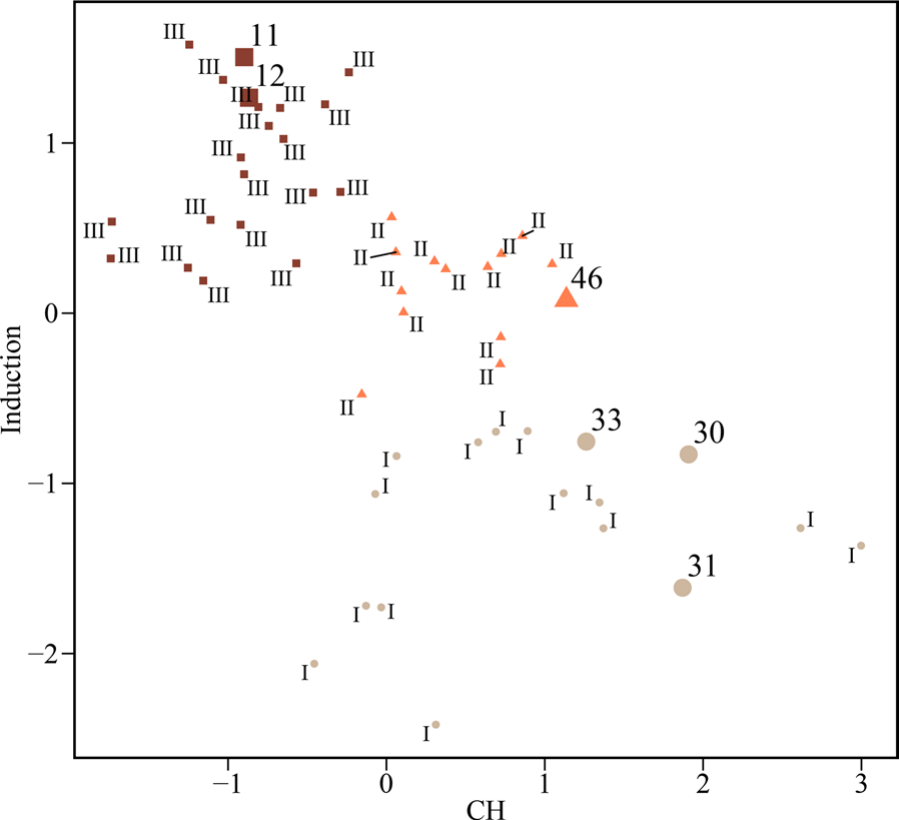
Performance validation of the clustering with Chl a only. The data used to make the clustering rules were expressed in Roman numerals to match the group numbers classified in Fig. 5. The validation data set was the same as the data used to validate the MLR and represented in Arabic numerals with their sample number. The samples were classified in cluster 1 (filled circle), cluster 2 (filled triangles), and cluster 3 (filled squares). CH Chl *a*

The average chlorophyll concentration of the groups with the most induced lipid was 0.11 pg cell^−1^, and most cells with chlorophyll below 0.15 pg cell^−1^ achieved high induction results (Additional file 5: Table S3). Since a gradual decrease in the chlorophyll contents after the nitrogen starvation is a natural progression, the protocol is very simple to apply: It is only necessary to wait until the chlorophyll concentration drops below the suggested threshold (< 0.15 pg cell^−1^). Besides the monotonic decreasing trend being simple to interpret, it has the advantage of easy implementation using sensors that can be purchased from the commercial market. But its application is restricted to microalgae with low chlorophyll contents since the physiological distinction between the groups in the range above the average chlorophyll content was ambiguous when the chlorophyll was solely used (Fig. 5e). However, this should not be all that limiting in the actual application as the goal is to distinguish the group with high lipid induction from those without. In Fig. 6, validation data (pointed by sample number) were classified and positioned in the plot. The cases that chlorophyll content was less than the suggested threshold (samples 11 and 12) got high induction results, and the samples above (samples 30, 31, 33, and 46) gave lower lipid induction results, as expected. Hence, the simple rule should be effective in deciding at the BPS whether microalgae have reached an internal state conductive to achieving a high lipid induction result at the LIS.

## Conclusion

It was shown that the lipid induction amount could be predicted accurately by means of the MLR model that includes the following proxy variables for the physiological status of the harvested cell: cell weight, total lipid contents, Chl *a*, and Fv/Fm. In particular, the Fv/Fm accounted for the largest portion in the variation and it had a maximum induction point at a value lower than its average. In the case that accurate prediction using all the regression variables is impractical due to measurement difficulties, it was recommended to harvest the cell when the chlorophyll content was below the 0.15 pg cell^−1^ to achieve a high induction result. Putting these results together, the stationary phase is generally the appropriate time to begin the lipid induction and this is indicated by the levels of chlorophyll and Fv/Fm falling low after the cell growth stops.

## Materials and methods

### Cell culture at the BPS

*Chlorella sorokiniana* HS1, a noble strain with halotolerance and lipid accumulation potential provided by Korea Research Institute of Bioscience and Biotechnology (KRIBB), was photo-autotrophically cultured [12]. In the BPS, modified BG11 medium including 0.75 g L^−1^ NaNO_3_, 0.2 g L^−1^ K_2_HPO_4_, and other BG11 components sterilized by a 0.45-μm bottle top filter (the RF 500 filter system, Sartorius Stedim Biotech, Germany) was used. Cells were inoculated in 500-mL Erlenmeyer baffled flasks containing 300 mL of the modified BG11 medium at the cell concentration of 1.00 × 10^6^ cells mL^−1^. The flasks were incubated in triplicate in a shaking incubator under 120 rpm, 150 μmol m^−2^ s^−1^ of fluorescent light. Filtered gas (2% CO_2_, v/v) was supplied to the culture at 100 mL min^−1^. In order to create a variety of cell statuses, cells were cultured at multiple temperatures (25, 30, and 35 °C) while maintaining the same above culture conditions and then transferred to the LIS while varying the harvesting time (Additional file 1: Table S1).

### Induction of lipid at the LIS

In order to satisfy the specification close to the developing industrial specification, harvested microalgal cells were centrifuged at 3000 rpm for 10 min to concentrate and adjust the initial cell concentration [2]. By doing so, the initial cell density in the LIS was maintained at 1.00 × 10^8^ cells mL^−1^. Collected cells were re-suspended to 250-mL Erlenmeyer flasks containing 100 mL of salted-modified BG11 medium. The salted-modified BG11 medium was made by adding 40 g L^−1^ NaCl and 1 g L^−1^ NaHCO_3_ to the modified BG11 medium. The cells were incubated for 24 h in a shaking incubator under 120 rpm, 150 μmol m^−2^ s^−1^ of fluorescent light, and 30 °C. Furthermore, cells grown at 30 °C in the BPS were additionally incubated under stress conditions for 48 h to observe changes in the morphology of lipid droplets. Gas was not supplied [2]; instead bicarbonate acted as an inorganic carbon source in the LIS, and there are many reports that bicarbonates have been used to increase lipid content in microalgae [10, 33, 34]. All the experiments were conducted at least three times. Averaged values of the results from the independent experiments were used for data analyses.

### Measurement of cell growth

Cell growth of *C. sorokiniana* HS1 in the broth was analyzed by measuring dry cell weight and cell density. In order to determine dry cell weight (DCW), samples were passed through circular glass filter paper (CF/G, 47 mm, Whatman), which was washed with deionized water (DW), and dried in a 70 °C oven overnight. For counting cells, an automated cell counter was used (Cellometer auto X4, Nexcelom, USA).

### Analysis of lipid

Total lipids were extracted by the Folch method [35] with some modifications. The freeze-dried cells were ground into powder and mixed with 10 mL of chloroform/methanol (2:1, v/v) mixture in a glass centrifuge tube. The mixture was incubated for 60 min at 100 °C, same temperatures used for fatty acid methyl esters (FAME) analysis [36], and vortexed for 10 min with 2.5 mL of 0.73% NaCl water solution. The solution was centrifuged at 4000 rpm for 10 min, and 5 mL of the bottom phase was transferred to pre-weighed aluminum weighing dishes. Chloroform phases in the weighing dishes were vaporized for 12 h in a fume hood at room temperature. Lipid percentage in microalgae was determined from the mass difference in the weighing dishes.

The lipid content (LC, pg cell^−1^) in microalgae, which refers to the amount of lipid per cell, was calculated by dividing the multiplication of lipid percentage and DCW by the cell density. As a result of lipid induction, the amount of lipid induced was assessed as the difference in LC between the stress-induced cell after the LIS and the harvested cell after the BPS. The mathematical expression is as follows:

1 $${\text{Amount}}\;{\text{of}}\;{\text{lipid}}\;{\text{induced }} = {\text{ LC}}_{\text{LIS}} \; - \;{\text{LC}}_{\text{BPS}}$$

where LC_LIS_ and LC_BPS_ indicate the lipid contents of stress-induced cell after the LIS and the lipid contents of harvested cell after the BPS, respectively.

### Analysis of carbohydrate

Total carbohydrate in cells was determined according to the colorimetric method using a UV/Vis spectrometer (DU 730, Beckman Coulter, USA), as described by Kang et al. [36]. Freeze-dried biomass was re-suspended in 10 mL of DW, and then 1 mL of samples was mixed with 1 mL of 5% (w/v) phenol solution. Then, 5 mL of highly concentrated sulfuric acid (95–98%) was added and incubated for 30 min at ambient temperature. After slight vortexing, optical density was measured at 470 nm using a UV/Vis spectrometer (DU 730, Beckman Coulter, USA). The amount of total carbohydrate was calculated from a standard which is made by glucose.

Starch was assayed by an enzymatic method using degradation of the starch to glucose with α-amylase and amyloglucosidase. Ground lyophilized cells were mixed with 10 mL of 80% (v/v) ethanol, and incubated at 80 °C for 60 min to remove interfering materials. Quantification of the starch percentage was conducted using a total starch assay kit (K-TSTA-100A, Megazyme, USA) according to the manufacturer’s instructions.

### Analysis of photosynthetic ability

Photosynthetic ability was investigated by measurement of Chl *a* and Fv/Fm. The concentration of chlorophyll *a* was analyzed by the dimethyl sulfoxide (DMSO) extraction method [37]. One milliliter of cultures of cells was centrifuged at 13,000 rpm for 10 min. The pellets were re-suspended in 1 mL DMSO and incubated at 70 °C for 30 min. Subsequently, the samples were centrifuged at 13,000 rpm for 10 min, and optical density was measured at 649 and 665 nm using the UV/Vis spectrometer (DU 730, Beckman Coulter, USA). The concentrations of chlorophyll *a* were calculated using Eq. (2) [38]:

2 $${\text{Chlorophyll}}\,{\text{a}}\left( {\upmu{\text{g}}\,{\text{mL}}^{ - 1} } \right) = 12.19 \times {\text{OD}}_{{665\,{\text{nm}}}} - 3.45 \times {\text{OD}}_{{649\,{\text{nm}}}}$$

The Chl *a* was calculated by dividing the concentration of chlorophyll *a* by the cell density. The chlorophyll fluorescence yield at 440 nm was measured in vivo with multi-color-PAM (Heinz Walz, Germany) to calculate Fv/Fm [39]. Prior to the fluorescence measurement, samples were diluted to 1.00 × 10^6^ cells mL^−1^, and 1.5 mL of the diluted samples were dark-adapted at 25 °C for 20 min.

### Microscopic observation with BODIPY 505/515

The lipophilic fluorescent dye BODIPY 505/515 was used as a vital stain to observe lipid bodies within live algal cells using the method described by Shin et al. [40] with some modifications. A 0.11 μg uL^−1^ stock solution of BODIPY 505/515 stock was prepared by dissolving the dye in DMSO and was stored in the dark at − 20 °C. Before the cell staining, the cell density of the samples was controlled to a concentration of 1.00 × 10^7^ cells mL^−1^, and 1.5 mL of the sample was transferred to a 2.0-mL amber-colored microcentrifuge tube. A frozen aliquot of the stock was thawed before use at room temperature, and 5 μL was added directly to the prepared algal suspensions. The algal–BODIPY suspension was vortexed for 10 min on a vortex mixer and then washed using DW. After the washing, the stained algae were used for microscopic observation.

Observations of neutral lipid bodies were performed by a Leica DM2500 microscope (Leica, Wetzlar, Germany), and photographs were taken with a Leica DFC 495 digital camera. The stained algal cells were observed under fluorescence microscopy with a 450–490 nm band excitation filter and a 515-nm-long emission filter.

### Development of linear predictive models

A MLR model was developed in the R system version 3.4.4. A polynomial structure containing the main effect, quadratic, and interaction terms was implemented. The model was employed for the amount of lipid induced in the form of Eq. (3):

3 $${\text{Amount}}\;{\text{of}}\;{\text{lipid}}\;{\text{induced}}\;\left( {\Delta {\text{pg}}\;{\text{cell}}^{ - 1} } \right)\; = \beta_{0} + \sum\limits_{i = 1}^{k} {\beta_{i} x_{i} } + \;\sum\limits_{i = 1}^{k} {\beta_{ii} x_{i}^{2} } + \;\sum\limits_{i < j} {\beta_{ij} x_{i} x_{j} }$$

where *x* and *β* represent a regressor and its coefficient, respectively. The subscripts *i* and *j* denote the proxy parameters for the physiological status of the harvested cell. The *β*_0_, the intercept term, was included to prevent biased residuals caused by forced modifications of the regression model.

Usually, bioprocess data are highly correlated and contain numerous noises; thus, the problem of multicollinearity and overfitting frequently occurs in bioprocess modeling. In general, for highly correlated variables such as chlorophyll and carotenoid, it is recommended to select representative variables before developing the model. Consequently, it is needed to exclude insignificant variables from candidate regressor variables. In order to identify the cell status to be included in the model, the method of all possible regressions is used as a variable selection method to prevent incorrect regression that can be caused by the order of variable selection [26]. The best regression model was selected on the basis of the Bayesian information criteria (BIC), which stand for the loss of information owing to the disagreement between the model and reality. In the hierarchical regression model, it is not recommended to remove a lower-order term from the model if the model includes higher-order terms of the same variables. Hence, if the higher-order terms were included in the selected model but the main effect of the same variable was excluded, the model was left out from the candidate group even if they had low BIC values.

The data set was split into a training set (90%) and test set (10%). Since uncentered data could introduce multicollinearity which leads to inflated variance among the regressor variables, all the data were pretreated by centering [41]. These inflated variances are problematic because these variances append little information to the model and make the calculated coefficients unreliable [42]. In particular, the multicollinearity problem is apt to occur in a regression model with interaction terms when the data are not centered [43]. Therefore, the model including variables with a high variance inflation factor (VIF) above 10 was not selected [42]. Statistical properties (autocorrelation, normality, homoscedasticity) were investigated to check the adequacy of the best model which had the lowest BIC value and satisfied the above constraints [44].

The relative importance of variables was calculated based on the *R*^2^ change in the model after a variable was excluded or included. Since the changes in *R*^2^ values can vary according to the order in which the variables change, the Lindeman, Merenda, and Gold (LMG) method using unweighted average values was used to account for the ordering problem [27].

### Clustering analysis

*K*-means clustering was performed in the R system version 3.4.4. The *K*-means clustering partitioned the *N* observations into *K* data sets so as to minimize the variance in the feature space of the observations within each cluster. The algorithm aimed at minimizing the within-cluster sum of squares represented by the following objective function (4):

4 $$\mathop {\arg \hbox{min} }\limits_{S} \sum\limits_{i = 1}^{K} {\sum\limits_{{x \in S_{i} }} {\left\| {x - \mu_{i} } \right\|^{2} } }$$

where *S*_*i*_, *K*, *x*, and *μ*_*i*_ represent a cluster, the total number of clusters, an observation, and each centroid in the cluster, respectively. The subscript *i* represents each cluster. The method of all possible was also introduced to determine the number of parameters used, the parameters to be used, and the number of clusters. In order to include the information about lipid induction in the clustering in all cases, the amount of induction was always used as a classification factor.

The longer the between-cluster distance is from the within-cluster distance, the better the classification. So, the silhouette score, which reflects both the distances at the same time, was used for the evaluation of clustering from the entire set [45]. Although all feature spaces are generally used to calculate the silhouette score, in this study, only the amount of lipid induced was used to maximize the separation capability for lipid accumulation even if the separation between the cell status is slightly decreased. In the case that different clusterings led to same scores with a same number of parameters used, those containing the variables with higher relative importance in the regression analysis were selected as the representative clusters.

## Supplementary information

**Additional file 1: Table S1.** Entire sampling data.

**Additional file 2: Figure S1.** Difference in the amount of lipid induced according to salt stress condition.

**Additional file 3: Figure S2.** Difference in the cell status according to culture temperature.

**Additional file 4: Table S2.** Silhouette score table.

**Additional file 5: Table S3.** Descriptive statistics of raw data on the selected factor for each cluster.

## Data Availability

All data generated or analyzed during this study are included in this published article.

## Abbreviations

BPS: biomass production stage
LIS: lipid induction stage
Chl *a*: chlorophyll *a* in a cell
Fv/Fm: maximum quantum yield of PSII
TAG: triacylglyceride
*C. sorokiniana* HS1: *Chlorella sorokiniana* HS1
BODIPY 505/515: 4,4-difluoro-1,3,5,7,-tetramethyl-4-bora-3a,4a-diaza-s-indacene
MLR: multiple linear regression
KRIBB: Korea research institute of bioscience and biotechnology
DCW: dry cell weight
DW: deionized water
FAME: fatty acid methyl ester
LC: lipid contents
DMSO: dimethyl sulfoxide
BIC: Bayesian information criteria
VIF: variance inflation factor
LMG: Lindeman, Merenda, and Gold
