# Supplementary material for: Identification of significant proxy variable for the physiological status affecting salt stress-induced lipid accumulation in Chlorella sorokiniana HS1
Source: Biotechnol Biofuels. 2019 Oct 12;12:242. doi: 10.1186/s13068-019-1582-9 (PMC6790037; doi:10.1186/s13068-019-1582-9)
Supplement: Supplementary file 1 — Additional file 1: Table S1. Entire sampling data. [file 13068_2019_1582_MOESM1_ESM.docx]

**Table S1. Entire sampling data**

| No.^a^ | Cultivation  Temperature  (°C) | Harvesting  Time  (d) | Cell  Weight  (pg cell^-1^) | Total  Lipid  (%) | Non-starch  Carbohydrate  (%) | Starch  (%) | Chlorophyll a  (pg cell^-1^) | Fv/Fm | Amount of  Lipid induced  (Δpg cell^-1^) | Purpose^b^ |
| --- | --- | --- | --- | --- | --- | --- | --- | --- | --- | --- |
| 1 | 30 | 2 | 11.7 ± 0.2 | 22.1 ± 0.3 | 18.8 ± 4.0 | 9.3 ± 0.1 | 0.19 ± 0.00 | 0.73 ± 0.00 | -0.25 ± 0.05 | Modeling |
| 2 | 30 | 3 | 9.7 ± 1.0 | 22.4 ± 1.0 | 16.0 ± 2.4 | 9.0 ± 1.0 | 0.17 ± 0.02 | 0.73 ± 0.01 | 0.60 ± 0.12 | Modeling |
| 3 | 30 | 4 | 10.2 ± 1.5 | 19.9 ± 1.0 | 16.1 ± 5.3 | 19.5 ± 3.4 | 0.13 ± 0.03 | 0.69 ± 0.01 | 3.07 ± 0.16 | Modeling |
| 4 | 30 | 5 | 8.0 ± 0.8 | 17.4 ± 0.2 | 21.1 ± 1.4 | 26.8 ± 1.1 | 0.12 ± 0.01 | 0.65 ± 0.02 | 4.06 ± 0.22 | Modeling |
| 5 | 30 | 6 | 9.2 ± 0.5 | 16.5 ± 0.7 | 17.4 ± 1.3 | 27.4 ± 0.3 | 0.11 ± 0.02 | 0.63 ± 0.00 | 4.20 ± 0.11 | Modeling |
| 6 | 30 | 7 | 13.7 ± 0.4 | 18.7 ± 0.8 | 16.6 ± 2.4 | 28.7 ± 0.4 | 0.13 ± 0.01 | 0.61 ± 0.01 | 3.97 ± 0.04 | Modeling |
| 7 | 30 | 8 | 11.4 ± 0.6 | 19.9 ± 0.7 | 20.8 ± 7.1 | 27.6 ± 1.1 | 0.11 ± 0.02 | 0.63 ± 0.00 | 3.35 ± 0.21 | Modeling |
| 8 | 30 | 9 | 13.5 ± 0.4 | 22.4 ± 0.8 | 12.8 ± 1.7 | 27.2 ± 0.4 | 0.12 ± 0.02 | 0.56 ± 0.00 | 4.19 ± 0.08 | Modeling |
| 9 | 30 | 10 | 13.1 ± 0.6 | 20.6 ± 1.1 | 15.3 ± 1.5 | 27.6 ± 1.1 | 0.14 ± 0.01 | 0.6 ± 0.00 | 4.22 ± 0.15 | Modeling |
| 10 | 30 | 11 | 13.4 ± 0.4 | 27.5 ± 0.4 | 17.9 ± 0.7 | 24.7 ± 0.4 | 0.08 ± 0.00 | 0.52 ± 0.00 | 4.65 ± 0.13 | Modeling |
| 11 | 30 | 12 | 14.6 ± 0.5 | 24.5 ± 1.6 | 12.0 ± 1.3 | 26.2 ± 1.1 | 0.11 ± 0.01 | 0.56 ± 0.00 | 4.56 ± 0.30 | Validation |
| 12 | 30 | 13 | 14.6 ± 0.7 | 27.9 ± 1.0 | 12.2 ± 0.6 | 23.9 ± 0.3 | 0.11 ± 0.00 | 0.54 ± 0.00 | 4.26 ± 0.09 | Validation |
| 13 | 30 | 14 | 16.7 ± 0.7 | 25.8 ± 1.7 | 16.3 ± 0.9 | 23.0 ± 0.1 | 0.14 ± 0.01 | 0.53 ± 0.00 | 3.58 ± 0.16 | Modeling |
| 14 | 25 | 2 | 9.9 ± 0.5 | 18.5 ± 0.2 | 13.1 ± 2.1 | 9.7 ± 0.4 | 0.14 ± 0.01 | 0.76 ± 0.00 | 0.19 ± 0.13 | Modeling |
| 15 | 25 | 4 | 10.9 ± 0.3 | 18.3 ± 0.3 | 13.3 ± 2.0 | 10.9 ± 0.0 | 0.27 ± 0.01 | 0.71 ± 0.00 | 1.35 ± 0.14 | Modeling |
| 16 | 25 | 6 | 8.9 ± 0.3 | 16.1 ± 0.2 | 17.8 ± 0.5 | 22.9 ± 0.4 | 0.19 ± 0.01 | 0.67 ± 0.00 | 3.09 ± 0.05 | Modeling |
| 17 | 25 | 8 | 9.4 ± 0.3 | 17.2 ± 0.4 | 20.3 ± 1.2 | 26.3 ± 0.4 | 0.17 ± 0.01 | 0.64 ± 0.00 | 3.41 ± 0.17 | Modeling |
| 18 | 25 | 10 | 10.2 ± 0.2 | 22.1 ± 0.2 | 12.8 ± 1.8 | 23.0 ± 0.1 | 0.15 ± 0.01 | 0.64 ± 0.00 | 4.45 ± 0.12 | Modeling |
| 19 | 25 | 12 | 10.8 ± 0.5 | 25.3 ± 0.4 | 17.3 ± 3.0 | 18.8 ± 2.7 | 0.10 ± 0.01 | 0.63 ± 0.00 | 4.39 ± 0.11 | Modeling |
| 20 | 25 | 14 | 15.5 ± 0.9 | 30.0 ± 0.7 | 19.6 ± 1.4 | 21.7 ± 0.3 | 0.11 ± 0.01 | 0.59 ± 0.00 | 3.84 ± 0.35 | Modeling |
| 21 | 35 | 2 | 7.4 ± 0.2 | 20.4 ± 0.9 | 21.4 ± 2.4 | 13.6 ± 1.7 | 0.38 ± 0.01 | 0.72 ± 0.00 | 1.04 ± 0.11 | Modeling |
| 22 | 35 | 4 | 10.7 ± 0.3 | 11.7 ± 0.3 | 9.0 ± 1.0 | 27.6 ± 0.5 | 0.16 ± 0.01 | 0.66 ± 0.00 | 2.13 ± 0.07 | Modeling |
| 23 | 35 | 6 | 12.2 ± 0.4 | 14.9 ± 0.3 | 17.9 ± 1.0 | 32.4 ± 0.1 | 0.15 ± 0.00 | 0.57 ± 0.00 | 3.59 ± 0.54 | Modeling |
| 24 | 35 | 8 | 12.5 ± 0.2 | 18.0 ± 0.3 | 22.4 ± 2.4 | 31.8 ± 0.1 | 0.18 ± 0.01 | 0.56 ± 0.00 | 3.15 ± 0.06 | Modeling |
| 25 | 35 | 10 | 13.1 ± 0.3 | 22.6 ± 0.5 | 15.0 ± 1.5 | 28.6 ± 0.7 | 0.11 ± 0.01 | 0.44 ± 0.01 | 3.71 ± 0.31 | Modeling |
| 26 | 35 | 12 | 14.1 ± 0.4 | 25.1 ± 1.0 | 8.4 ± 1.8 | 26.4 ± 0.4 | 0.09 ± 0.00 | 0.41 ± 0.00 | 2.95 ± 0.03 | Modeling |
| 27 | 35 | 14 | 14.4 ± 0.7 | 29.2 ± 0.2 | 17.1 ± 0.9 | 23.4 ± 0.2 | 0.08 ± 0.00 | 0.36 ± 0.00 | 3.04 ± 0.31 | Modeling |
| 28 | 25 | 5 | 9.8 ± 0.4 | 21.8 ± 0.0 | 16.0 ± 0.7 | 8.8 ± 0.6 | 0.36 ± 0.01 | 0.71 ± 0.00 | 1.17 ± 0.11 | Modeling |
| 29 | 35 | 5 | 10.4 ± 0.3 | 19.8 ± 0.2 | 19.9 ± 0.2 | 16.9 ± 0.1 | 0.27 ± 0.01 | 0.69 ± 0.00 | 1.17 ± 0.12 | Modeling |
| 30 | 25 | 7 | 12.2 ± 0.9 | 18.7 ± 0.1 | 28.4 ± 6.2 | 21.4 ± 0.3 | 0.31 ± 0.02 | 0.68 ± 0.00 | 1.70 ± 0.35 | Validation |
| 31 | 35 | 7 | 15.2 ± 0.6 | 18.3 ± 1.3 | 22.6 ± 2.2 | 28.8 ± 0.2 | 0.30 ± 0.01 | 0.66 ± 0.00 | 0.74 ± 0.09 | Validation |
| 32 | 25 | 9 | 13.1 ± 0.6 | 23.0 ± 0.2 | 24.8 ± 1.6 | 26.4 ± 0.0 | 0.22 ± 0.01 | 0.64 ± 0.00 | 2.35 ± 0.28 | Modeling |
| 33 | 35 | 9 | 15.7 ± 0.6 | 17.9 ± 0.3 | 24.3 ± 0.8 | 29.8 ± 0.5 | 0.26 ± 0.02 | 0.64 ± 0.00 | 1.79 ± 0.11 | Validation |
| 34 | 25 | 11 | 15.7 ± 0.8 | 23.1 ± 0.3 | 33.6 ± 1.6 | 24.3 ± 0.2 | 0.22 ± 0.01 | 0.64 ± 0.00 | 3.05 ± 0.09 | Modeling |
| 35 | 35 | 11 | 17.6 ± 0.6 | 20.1 ± 0.1 | 26.9 ± 11.3 | 31.3 ± 0.2 | 0.21 ± 0.01 | 0.57 ± 0.00 | 1.79 ± 0.13 | Modeling |
| 36 | 25 | 13 | 16.7 ± 0.2 | 26.4 ± 0.4 | 27.3 ± 6.8 | 23.3 ± 0.0 | 0.18 ± 0.00 | 0.60 ± 0.00 | 2.72 ± 0.07 | Modeling |
| 37 | 35 | 13 | 19.0 ± 0.6 | 23.3 ± 0.1 | 14.9 ± 0.7 | 31.4 ± 0.0 | 0.18 ± 0.01 | 0.52 ± 0.00 | 1.69 ± 0.08 | Modeling |
| 38 | 30 | 8 | 12.8 ± 0.5 | 18.3 ± 0.4 | 27.4 ± 3.0 | 28.4 ± 0.2 | 0.25 ± 0.01 | 0.65 ± 0.00 | 3.07 ± 0.24 | Modeling |
| 39^*^ | 30 | 8 | 12.0 ± 0.6 | 26.3 ± 0.8 | 34.0 ± 2.4 | 19.1 ± 0.1 | 0.09 ± 0.01 | 0.62 ± 0.00 | 3.39 ± 0.42 | Modeling |
| 40 | 25 | 10 | 16.3 ± 1.3 | 21.2 ± 0.5 | 25.2 ± 0.9 | 25.3 ± 0.1 | 0.22 ± 0.01 | 0.63 ± 0.00 | 3.14 ± 0.52 | Modeling |
| 41 | 35 | 10 | 18.1 ± 1.0 | 20.6 ± 0.2 | 20.5 ± 1.7 | 30.7 ± 0.1 | 0.22 ± 0.01 | 0.61 | 2.54 ± 0.46 | Modeling |
| 42 | 30 | 10 | 15.4 ± 0.5 | 22.0 ± 0.4 | 18.9 ± 6.3 | 26.6 ± 0.2 | 0.23 ± 0.01 | 0.63 | 3.27 ± 0.50 | Modeling |
| 43^*^ | 30 | 10 | 12.9 ± 0.9 | 36.1 ± 1.2 | 22.7 ± 2.3 | 15.6 ± 0.3 | 0.05 ± 0.01 | 0.58 | 3.37 ± 0.27 | Modeling |
| 44 | 30 | 12 | 16.5 ± 0.8 | 24.6 ± 0.3 | 23.1 ± 1.6 | 25.9 ± 0.5 | 0.20 ± 0.01 | 0.57 | 3.03 ± 0.14 | Modeling |
| 45^*^ | 30 | 12 | 14.3 ± 0.7 | 38.1 ± 0.4 | 18.2 ± 1.3 | 15.8 ± 0.3 | 0.05 ± 0.00 | 0.56 | 3.11 ± 0.09 | Modeling |
| 46 | 25 | 12 | 17.2 ± 0.9 | 21.7 ± 0.1 | 25.8 ± 0.3 | 23.7 ± 0.2 | 0.25 ± 0.02 | 0.63 | 2.81 ± 0.55 | Validation |
| 47 | 30 | 12 | 18.2 ± 0.2 | 22.1 ± 0.5 | 22.4 ± 0.4 | 24.9 ± 0.2 | 0.25 ± 0.01 | 0.59 | 1.42 ± 0.44 | Modeling |
| 48 | 35 | 12 | 16.4 ± 1.1 | 16.8 ± 1.0 | 22.9 ± 0.4 | 27.2 ± 0.2 | 0.22 ± 0.01 | 0.61 | 1.86 ± 0.34 | Modeling |
| 49 | 25 | 14 | 17.8 ± 1.1 | 22.6 ± 0.3 | 27.8 ± 3.6 | 22.9 ± 0.1 | 0.18 ± 0.02 | 0.59 | 2.87 ± 0.49 | Modeling |
| 50 | 30 | 14 | 20.1 ± 1.1 | 22.7 ± 0.2 | 23.9 ± 1.0 | 24.2 ± 0.3 | 0.16 ± 0.02 | 0.55 | 0.61 ± 0.29 | Modeling |
| 51 | 35 | 14 | 17.8 ± 0.9 | 20.3 ± 0.9 | 25.2 ± 1.2 | 25.4 ± 0.1 | 0.17 ± 0.02 | 0.51 | 1.41 ± 0.21 | Modeling |
| 52 | 30 | 9 | 15.4 ± 0.2 | 23.0 ± 1.9 | 27.1 ± 1.3 | 27.4 ± 1.0 | 0.23 ± 0.01 | 0.65 | 1.87 ± 0.27 | Modeling |

^a^Microalgae were cultivated with the modified BG11 medium. The samples marked by asterisk(*) were cultivated in a medium that initial nitrogen concentration is reduced(half) than the original modified BG11 medium.

^b^About 10% (6 samples) of the entire samples were used to validate the developed model.
