# Supplementary material for: Identification of significant proxy variable for the physiological status affecting salt stress-induced lipid accumulation in Chlorella sorokiniana HS1
Source: Biotechnol Biofuels. 2019 Oct 12;12:242. doi: 10.1186/s13068-019-1582-9 (PMC6790037; doi:10.1186/s13068-019-1582-9)
Supplement: Supplementary file 2 — Additional file 2: Figure S1. Difference in the amount of lipid induced according to salt stress condition. [file 13068_2019_1582_MOESM2_ESM.docx]

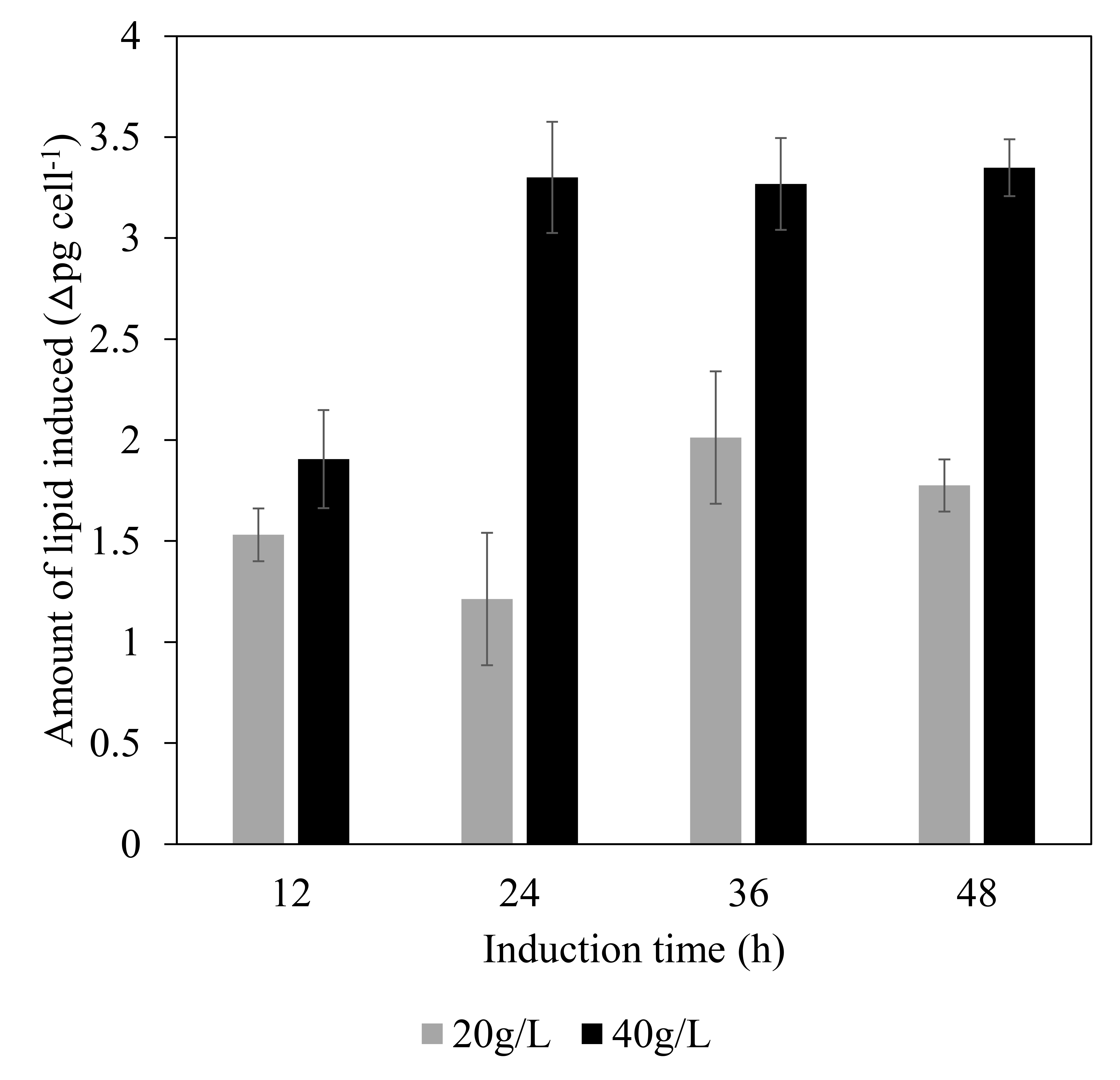


**Figure S1.** Difference in the amount of lipid induced according to salt stress condition. Cells were cultivated for 7 days before lipid induction.
