# Supplementary material for: Identification of significant proxy variable for the physiological status affecting salt stress-induced lipid accumulation in Chlorella sorokiniana HS1
Source: Biotechnol Biofuels. 2019 Oct 12;12:242. doi: 10.1186/s13068-019-1582-9 (PMC6790037; doi:10.1186/s13068-019-1582-9)
Supplement: Supplementary file 3 — Additional file 3: Figure S2. Difference in the cell status according to culture temperature. [file 13068_2019_1582_MOESM3_ESM.docx]

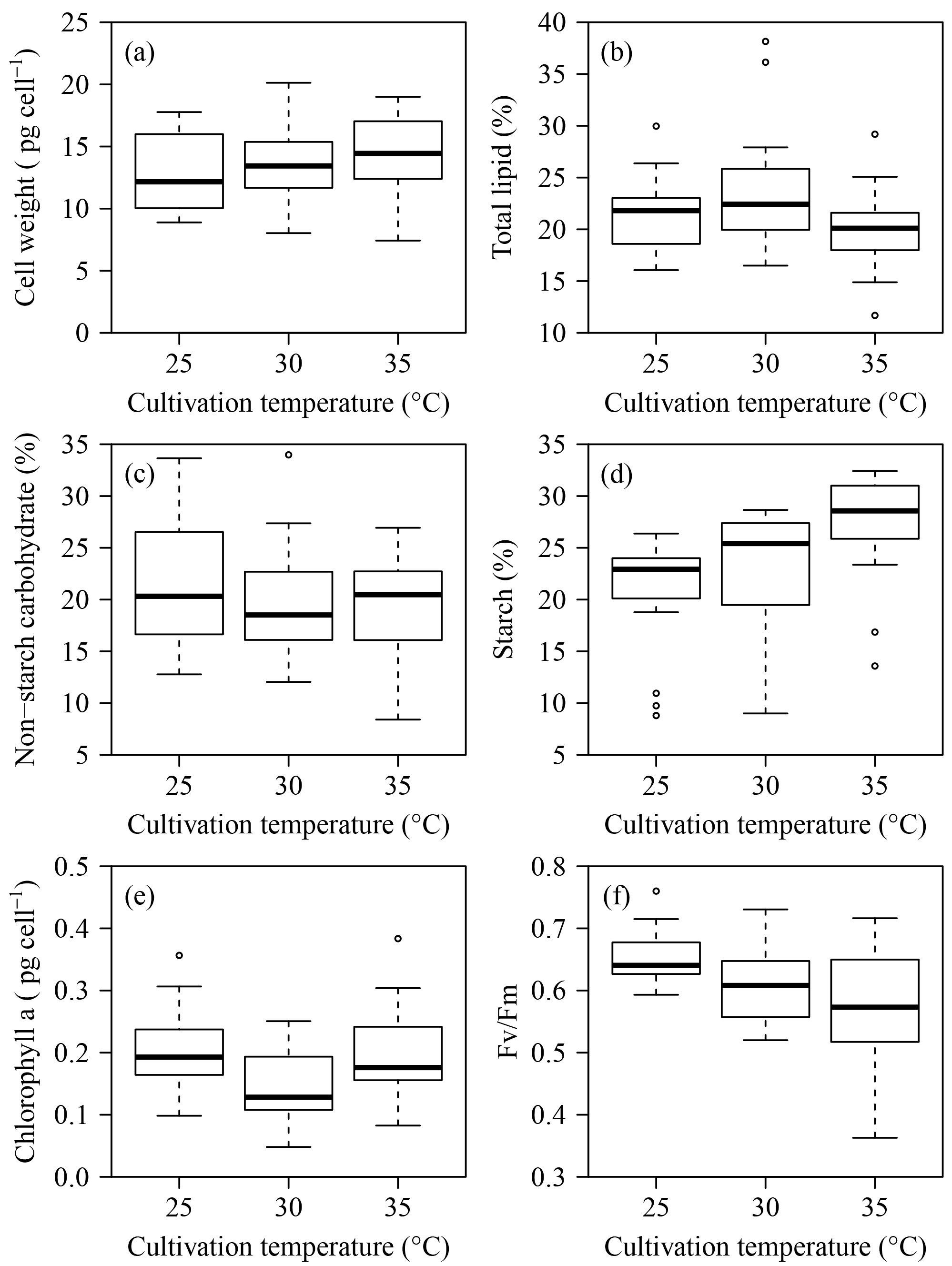


**Figure S2.** Difference in the cell status according to culture temperature. Cell weight (a), total lipid content (b), non-starch carbohydrate content (c), starch content (d), Chl a (e), and Fv/Fm (f).
