# Supplementary material for: Identification of significant proxy variable for the physiological status affecting salt stress-induced lipid accumulation in Chlorella sorokiniana HS1
Source: Biotechnol Biofuels. 2019 Oct 12;12:242. doi: 10.1186/s13068-019-1582-9 (PMC6790037; doi:10.1186/s13068-019-1582-9)
Supplement: Supplementary file 4 — Additional file 4: Table S2. Silhouette score table. [file 13068_2019_1582_MOESM4_ESM.docx]

**Table S2. Silhouette score table**^a^

| Number of intrinsic condition | Number of cluster  Intrinsic condition^b^ | 3 | 4 | 5 | 6 | 7 | 8 | 9 | 10 |
| --- | --- | --- | --- | --- | --- | --- | --- | --- | --- |
| 3 | L, CH, FV | 0.036 | 0.012 | -0.128 | -0.173 | -0.250 | -0.160 | -0.189 | -0.185 |
|  | CW, CH, FV | 0.251^c^ | 0.000 | -0.026 | 0.001 | -0.110 | -0.121 | -0.179 | -0.134 |
|  | CW, L, FV | 0.053 | 0.002 | -0.128 | -0.144 | -0.081 | -0.199 | -0.222 | -0.281 |
|  | CW, L, CH | 0.251^c^ | 0.008 | -0.185 | -0.228 | -0.178 | -0.151 | -0.129 | -0.137 |
| 2 | CW, L | 0.178 | 0.008 | -0.176 | -0.042 | -0.148 | -0.175 | -0.235 | -0.247 |
|  | CW, CH | 0.237 | 0.174 | -0.024 | 0.022 | -0.033 | -0.042 | -0.114 | -0.107 |
|  | CW, FV | 0.133 | -0.020 | -0.103 | -0.143 | -0.147 | -0.178 | -0.148 | -0.140 |
|  | L, CH | 0.151 | 0.156 | 0.030 | -0.158 | -0.137 | -0.140 | -0.177 | -0.218 |
|  | L, FV | 0.163 | 0.022 | -0.088 | -0.153 | -0.121 | -0.242 | -0.203 | -0.204 |
|  | CH, FV | 0.274 | -0.015 | -0.066 | 0.046 | -0.053 | -0.016 | -0.009 | -0.139 |
| 1 | CW | 0.222 | 0.150 | 0.048 | -0.003 | 0.009 | -0.040 | -0.053 | -0.129 |
|  | L | 0.155 | -0.053 | -0.067 | 0.045 | -0.109 | -0.049 | -0.118 | -0.127 |
|  | CH | 0.358 | 0.233 | 0.221 | 0.140 | 0.102 | 0.069 | 0.034 | 0.064 |
|  | FV | 0.270 | 0.007 | -0.004 | -0.019 | 0.065 | 0.049 | 0.091 | 0.062 |

^a^The silhouette score was calculated solely for the amount of lipid-induced, and the ability to classify for other physiological conditions were not included in the score.

^b^Abbreviation: CW, cell weight; L, total lipid content; CH, Chl *a*; FV, Fv/Fm. In order to include the information about lipid induction in the clustering all the time, the amount of induction was always used as a classification factor.

^c^The combination (number of cluster: 3) with CW, L, and FV also had a higher silhouette score as much as the classification (number of cluster: 3) with CW, CH, and FV. As shown in the relative importance of each parameter, the model including Fv/Fm was selected as the representative model since it was found that the Fv/Fm can account for the variation in induction more than the total lipid.
