# Supplementary material for: Identification of significant proxy variable for the physiological status affecting salt stress-induced lipid accumulation in Chlorella sorokiniana HS1
Source: Biotechnol Biofuels. 2019 Oct 12;12:242. doi: 10.1186/s13068-019-1582-9 (PMC6790037; doi:10.1186/s13068-019-1582-9)
Supplement: Supplementary file 5 — Additional file 5: Table S3. Descriptive statistics of raw data on the selected factor for each cluster. [file 13068_2019_1582_MOESM5_ESM.docx]

**Table S3. Descriptive statistics of raw data on the selected factor for each cluster**

| Intrinsic condition^a^ | Group | Factor | Min | Max | Mean | Median | SD^a^ |
| --- | --- | --- | --- | --- | --- | --- | --- |
| CW, CH, FV | Cluster 1 | CW | 12.8 | 20.1 | 16.7 | 16.6 | 2.0 |
|  |  | CH | 0.16 | 0.25 | 0.21 | 0.22 | 0.03 |
|  |  | FV | 0.51 | 0.65 | 0.60 | 0.60 | 0.04 |
|  |  | Induction | 0.61 | 3.27 | 2.29 | 2.44 | 0.79 |
|  |  |  |  |  |  |  |  |
|  | Cluster 2 | CW | 7.4 | 11.7 | 9.9 | 9.9 | 1.2 |
|  |  | CH | 0.14 | 0.38 | 0.24 | 0.19 | 0.09 |
|  |  | FV | 0.66 | 0.76 | 0.71 | 0.71 | 0.03 |
|  |  | Induction | -0.25 | 3.09 | 1.16 | 1.17 | 1.00 |
|  |  |  |  |  |  |  |  |
|  | Cluster 3 | CW | 8.0 | 16.7 | 12.4 | 12.9 | 2.2 |
|  |  | CH | 0.05 | 0.18 | 0.11 | 0.11 | 0.03 |
|  |  | FV | 0.36 | 0.69 | 0.57 | 0.59 | 0.08 |
|  |  | Induction | 2.95 | 4.65 | 3.70 | 3.59 | 0.52 |
|  |  |  |  |  |  |  |  |
| CH, FV | Cluster 1 | CH | 0.13 | 0.25 | 0.20 | 0.20 | 0.03 |
|  |  | FV | 0.51 | 0.69 | 0.61 | 0.61 | 0.05 |
|  |  | Induction | 0.61 | 3.41 | 2.45 | 2.72 | 0.78 |
|  |  |  |  |  |  |  |  |
|  | Cluster 2 | CH | 0.14 | 0.38 | 0.25 | 0.27 | 0.09 |
|  |  | FV | 0.69 | 0.76 | 0.72 | 0.72 | 0.02 |
|  |  | Induction | -0.25 | 1.35 | 0.75 | 1.04 | 0.60 |
|  |  |  |  |  |  |  |  |
|  | Cluster 3 | CH | 0.05 | 0.15 | 0.11 | 0.11 | 0.03 |
|  |  | FV | 0.36 | 0.65 | 0.56 | 0.59 | 0.08 |
|  |  | Induction | 2.95 | 4.65 | 3.78 | 3.77 | 0.51 |
|  |  |  |  |  |  |  |  |
| CH | Cluster 1 | CH | 0.14 | 0.38 | 0.23 | 0.22 | 0.07 |
|  |  | Induction | -0.25 | 1.87 | 1.14 | 1.26 | 0.64 |
|  |  |  |  |  |  |  |  |
|  | Cluster 2 | CH | 0.16 | 0.25 | 0.20 | 0.20 | 0.03 |
|  |  | Induction | 2.13 | 3.41 | 2.91 | 3.05 | 0.37 |
|  |  |  |  |  |  |  |  |
|  | Cluster 3 | CH | 0.05 | 0.15 | 0.11 | 0.11 | 0.03 |
|  |  | Induction | 2.95 | 4.65 | 3.74 | 3.71 | 0.53 |

^a^Abbreviation: CW, cell weight; L, total lipid content; CH, Chl *a*; FV, Fv/Fm; Induction, amount of lipid induced (Δpg cell^-1^); SD, standard deviation.
